# Neonatal outcomes of maternal SARS-CoV-2 infection in the UK: a prospective cohort study using active surveillance

**DOI:** 10.1038/s41390-023-02527-z

**Published:** 2023-03-10

**Authors:** Shohaib Ali, Helen Mactier, Alessandra Morelli, Madeleine Hurd, Anna Placzek, Marian Knight, Shamez N. Ladhani, Elizabeth S. Draper, Don Sharkey, Cora Doherty, Jennifer J. Kurinczuk, Maria A. Quigley, Chris Gale

**Affiliations:** 1 School of Public Health, Faculty of Medicine, Imperial College London, London, UK; 2 Princess Royal Maternity and the University of Glasgow, Glasgow, UK; 3 NIHR Policy Research Unit in Maternal and Neonatal Health and Care, National Perinatal Epidemiology Unit, Nuffield Department of Population Health, University of Oxford, Oxford, UK; 4 Paediatric Infectious Diseases and Vaccinology, St. George’s University of London, London, UK; 5 Department of Health Sciences, Centre for Medicine, University of Leicester, University Road, Leicester, UK; 6 Centre for Perinatal Research, School of Medicine, University of Nottingham, Nottingham, UK; 7 University Hospital of Wales, Cardiff, UK; 8 School of Public Health, Faculty of Medicine, Imperial College London, Chelsea and Westminster Campus, 369 Fulham Road, London, SW10 9NH UK

## Abstract

**Background:**

Newborns may be affected by maternal SARS-CoV-2 infection during pregnancy. We aimed to describe the epidemiology, clinical course and short-term outcomes of babies admitted to a neonatal unit (NNU) following birth to a mother with confirmed SARS-CoV-2 infection within 7 days of birth.

**Methods:**

This is a UK prospective cohort study; all NHS NNUs, 1 March 2020 to 31 August 2020. Cases were identified via British Paediatric Surveillance Unit with linkage to national obstetric surveillance data. Reporting clinicians completed data forms. Population data were extracted from the National Neonatal Research Database.

**Results:**

A total of 111 NNU admissions (1.98 per 1000 of all NNU admissions) involved 2456 days of neonatal care (median 13 [IQR 5, 34] care days per admission). A total of 74 (67%) babies were preterm. In all, 76 (68%) received respiratory support; 30 were mechanically ventilated. Four term babies received therapeutic hypothermia for hypoxic ischaemic encephalopathy. Twenty-eight mothers received intensive care, with four dying of COVID-19. Eleven (10%) babies were SARS-CoV-2 positive. A total of 105 (95%) babies were discharged home; none of the three deaths before discharge was attributed to SARS-CoV-2.

**Conclusion:**

Babies born to mothers with SARS-CoV-2 infection around the time of birth accounted for a low proportion of total NNU admissions over the first 6 months of the UK pandemic. Neonatal SARS-CoV-2 was uncommon.

**Study registration:**

ISRCTN60033461; protocol available at http://www.npeu.ox.ac.uk/pru-mnhc/research-themes/theme-4/covid-19.

**Impact:**

- Neonatal unit admissions of babies born to mothers with SARS-CoV-2 infection comprised only a small proportion of total neonatal admissions in the first 6 months of the pandemic.
- A high proportion of babies requiring neonatal admission who were born to mothers with confirmed SARS-CoV-2 infection were preterm and had neonatal SARS-CoV-2 infection and/or other conditions associated with long-term sequelae.
- Adverse neonatal conditions were more common in babies whose SARS-CoV-2-positive mothers required intensive care compared to those whose SARS-CoV-2-positive mothers who did not.

## Introduction

Although serious infection with SARS-CoV-2 is uncommon in children, maternal SARS-CoV-2 infection around the time of birth is associated with adverse outcomes, including preterm birth^1,2^ and neonatal infection.^3–5^ Swedish data demonstrate a higher risk of preterm birth, neonatal unit (NNU) admission and neonatal respiratory conditions following birth to a mother with SARS-CoV-2 at any time in pregnancy.^6^ Although studies show adverse pregnancy outcomes increased in those mothers with severe/critical disease,^7^ only limited data describe neonatal clinical courses and outcomes. Accumulating data indicate that the Alpha and Delta variants of SARS-CoV-2 may lead to more severe maternal COVID-19;^1^ for comparative purposes, it is important to establish neonatal impacts of the initial wave of predominantly ‘wildtype’ SARS-CoV-2 infection.

We aimed to describe the presentation, clinical course and short-term outcomes of babies born in the first wave of the pandemic to a mother with confirmed SARS-CoV-2 infection within 7 days of birth and who required NNU admission. Neonatal outcomes were examined by the severity of maternal COVID-19.

## Methods

### Study design and procedures

This was a national prospective cohort study using the British Paediatric Surveillance Unit (BPSU).^8^ From 1 April 2020, senior paediatricians in all 155 hospital trusts and health boards with their associated 190 NNUs in the UK received a weekly electronic BPSU reporting card asking them to notify any babies born to mothers with laboratory-confirmed SARS-CoV-2 infection in the 7 days before or after birth, and who required admission to an NNU between 1 March 2020 to 30 August 2020. This did not include routine newborn care provided on the postnatal ward by neonatal health professionals. An additional monthly card asked for confirmation both that all eligible babies had been reported and that zero reports were accurate (active negative surveillance). To maximise case ascertainment, the UK Obstetric Surveillance System (UKOSS) study of COVID-19 admission in pregnancy was also used to identify and link babies born to women with laboratory-confirmed SARS-CoV-2 infection using hospital site, date and time of delivery. Following a BPSU report, notifying clinicians were asked to complete a data collection form with details of the pregnancy, baby characteristics, neonatal management and outcomes. Data collection was supported by hospital-based research nurses from the UK’s National Institute of Health Research (NIHR) Clinical Research Network following the study’s adoption as an urgent public health priority study.

Cases identified were linked to routinely recorded neonatal clinical data held in the National Neonatal Research Database (NNRD) to describe neonatal treatments, clinical course and outcomes.^8^ Neonatal deaths were cross-checked with national perinatal mortality surveillance data from the Mother and Babies, Reducing Risk through Audits and Confidential Enquires across the UK (MBRRACE-UK) collaboration.^9^ Neonatal SARS-CoV-2 infection was cross-checked with national testing data from public health organisations as described previously.^4^

UK SARS-CoV-2 testing policy amongst pregnant women and babies evolved during the study. Initially, only symptomatic women and babies were tested. Routine screening of all obstetric admissions was recommended by the Royal College of Obstetricians and Gynaecologists on 29 May 2020 and neonatal testing was recommended for symptomatic babies of mothers with a SARS-CoV-2 infection; testing of asymptomatic babies varied.^10^ Confirmation of neonatal SARS-CoV-2 infection required at least two positive samples, including one at least 72 h after birth.^10^ UK policy was that well babies of SARS-CoV-2-infected mothers should be cared for alongside their mothers in the postnatal ward.

This report presents the characteristics and short-term outcomes for babies born to a mother with confirmed SARS-CoV-2 infection in the 7 days before or after birth between 1 March and 31 August 2020 and for whom complete data had been received by 14 January 2021.

Maternal admission criteria were based on locally defined clinical indications.^11^ Neonatal intensive care was defined using the British Association of Perinatal Medicine criteria.^12^ Denominator data for total NNU admissions over the study period were obtained from the NNRD and data describing maternal SARS-CoV-2 infections were taken from the UKOSS which recorded all pregnant women in hospitals with confirmed SARS-CoV-2 infection.^11^

### Parent, patient and public involvement

Parents, patients and the public were consulted during the design of the study and presentation of the findings through the MBRRACE-UK and the NIHR Policy Research Unit in the Maternal and Neonatal Care third-sector stakeholder groups comprising representatives from all the relevant national mother and baby charities in the UK.

### Statistical analysis

Descriptive statistics are presented as means with standard deviations and medians with interquartile ranges.

## Results

**Fig. 1:**
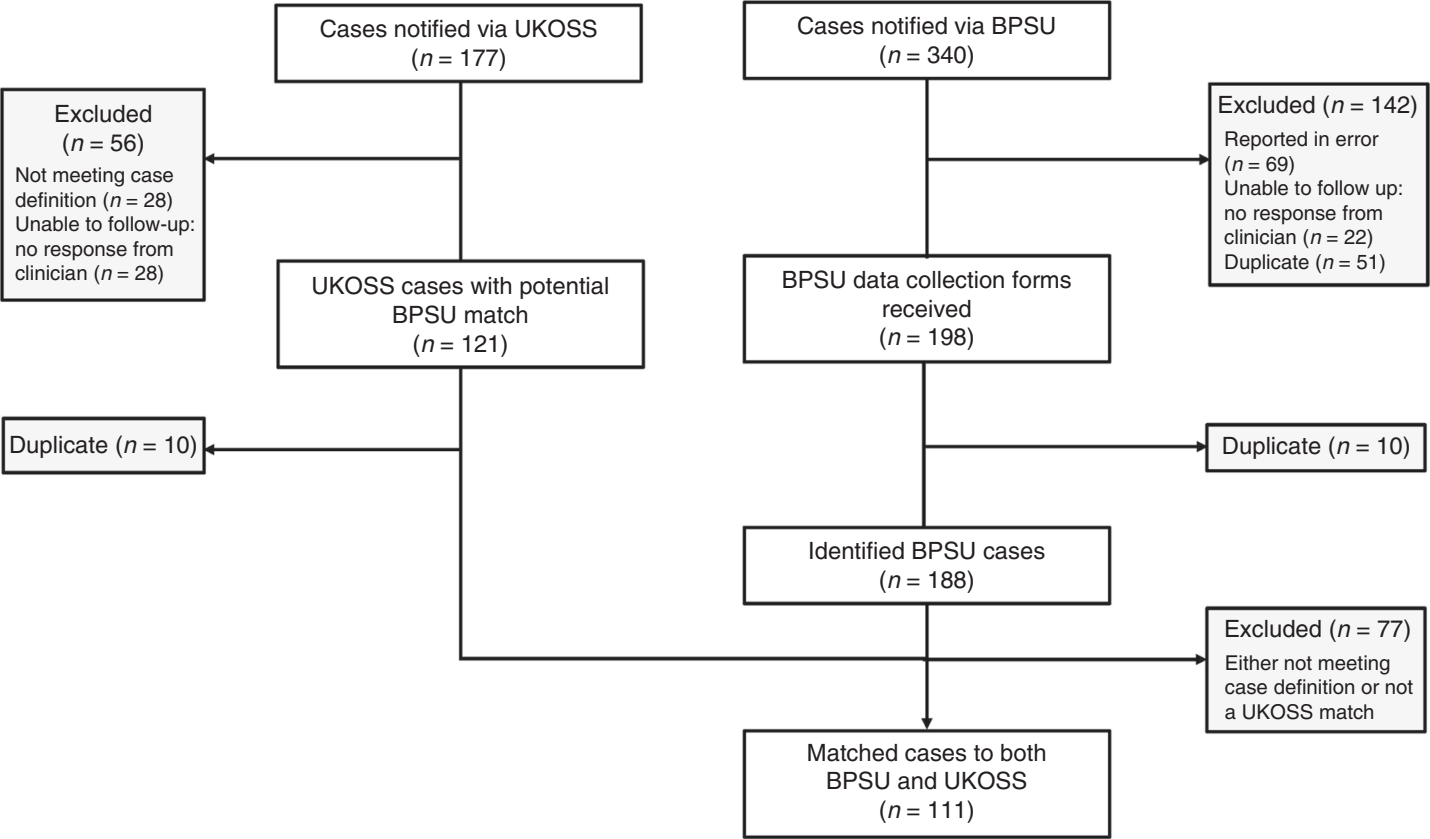
Flowchart of case selection for the study period 1st March 2020 to 31st August 2020. This flowchart describes how cases were selected including the linkage between BPSU data and UKOSS data.

There were 309,518 live births during the study period; 1019 babies were born in the UK to mothers known to have SARS-CoV-2 infection within 7 days of birth during the study period (634 symptomatic mothers, 385 asymptomatic mothers). All were singleton pregnancies. Monthly BPSU card returns were received from between 87.0% and 91.3% of 4091 UK paediatricians each month; 340 potentially eligible babies were reported through the BPSU system and 177 through UKOSS surveillance (Fig. 1). After de-duplication and exclusion of ineligible babies, 111/1019 (10.9%) were admitted to an NNU following maternal SARS-CoV-2 infection around birth.

**Fig. 2:**
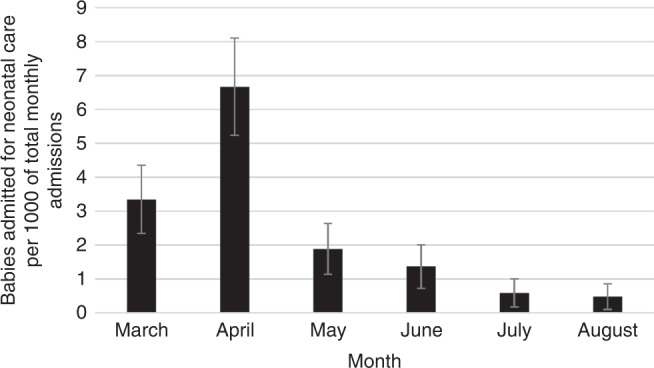
Proportion of babies admitted for neonatal care following maternal coronavirus. Babies admitted for neonatal care following maternal coronavirus as a proportion per 1,000 of total monthly neonatal admissions between March 2020 to 31st August 2020.

During the study period, 56,179 babies were admitted to an NNU for any reason. Therefore, overall 2 per 1000 (95% CI 1.6–2.4) neonatal admissions were of babies born to mothers with SARS-CoV-2 infection around the time of birth. The highest proportions were in March and April 2020 (3.4 and 6.7 per 1000 admissions, respectively) (Fig. 2). 74/111 babies (67%) were born preterm (<37 weeks’ gestation), including 26 (23%) born very preterm (<32 weeks’ gestation). Over the study period, 6 per 1000 (95% CI 4.4–9.4) very preterm neonatal admissions were babies born to mothers with perinatal SARS-CoV-2 infection. Among babies admitted for neonatal care, 55/111 babies (50%) were born to mothers from non-white ethnic groups (Table 1).

**Table 1:** Maternal and birth characteristics for babies admitted for neonatal care following maternal SARS-CoV-2 infection around the time of birth in the UK by maternal intensive care status.

	Neonatal care following maternal SARS-CoV-2 around the time of birth	Neonatal care following maternal intensive care	Neonatal care following maternal non-intensive care
Number	111	28^a^	76^a^
Maternal age			
<35 years	62 (55%)	13 (46%)	49 (65%)
≥35 years	42 (38%)	15 (54%)	27 (36%)
Missing	7 (6%)	0 (0%)	0 (0%)
Maternal BMI			
Normal	33 (30%)	6 (21%)	27 (35%)
Overweight	32 (29%)	14 (50%)	18 (23%)
Obese	35 (32%)	7 (25%)	28 (37%)
Missing	11 (10%)	1 (3%)	3 (4%)
Maternal ethnicity			
White	48 (43%)	11 (39%)	37 (49%)
Asian	25 (23%)	5 (18%)	20 (26%)
Black	25 (23%)	10 (36%)	15 (20%)
Other	2 (2%)	0 (0%)	2 (3%)
Mixed	3 (3%)	2 (7%)	1 (1%)
Missing	8 (7%)	0 (0%)	0 (0%)
Antenatal steroids given			
Eligible (<34 weeks)	37	18	19
Full	18 (49%)	8 (44%)	10 (53%)
Partial	16 (43%)	7 (39%)	9 (47%)
Not indicated	3 (8%)	3 (17%)	0 (0%)
Magnesium sulfate given			
Eligible (<30 weeks)	11	8	3
Yes	10 (90%)	7 (88%)	3 (100%)
No	1 (10%)	1 (12%)	0 (0%)
Not indicated			
Mode of birth			
Unassisted vaginal birth	18 (16%)	0 (0%)	18 (24%)
Instrumental vaginal birth	6 (5%)	1 (4%)	5 (7%)
Caesarean birth, maternal SARS-CoV-2	33 (30%)	25 (89%)	8 (11%)
Caesarean birth, unrelated to SARS-CoV-2	42 (38%)	2 (7%)	40 (53%)
Missing	12 (11%)	0 (0%)	2 (3%)
Gestational age at birth			
22–27 weeks	2 (2%)	1 (4%)	1 (1%)
28–31 weeks	24 (22%)	11 (39%)	9 (12%)
32–36 weeks	48 (43%)	14 (50%)	33 (43%)
≥37 weeks	37 (33%)	2 (7%)	33 (43%)

^a^Maternal intensive care status was missing for 7 babies.

Severity of illness from SARS-CoV-2 infection among the mothers of the 111 admitted infants varied: 28/104 (27%) mother’s required intensive care, of whom four died from SARS-CoV-2; maternal intensive care data were missing for seven cases.

Forty of 111 (36%) babies required neonatal intensive care; 76 (68%) were treated with some form of respiratory support of whom 30 received mechanical ventilation. Four term babies received therapeutic hypothermia for hypoxic ischaemic encephalopathy (Table 2). Eleven (10%) babies had confirmed neonatal SARS-CoV-2. In total, babies born to mothers with SARS-CoV-2 infection around birth received 2456 days of neonatal care (median 13 days [IQR 5, 34] per baby), including 295 days of intensive care (median 0 days [IQR 0, 2] per baby). At the end of follow-up, 105 (95%) babies had been discharged home and two were still admitted to an NNU. Three babies died before discharge from the NNU; outcome data were missing for one baby. In no instance was the cause of death attributed to neonatal or maternal SARS-CoV-2 infection.

**Table 2:** Characteristics, treatments and outcomes of babies admitted for neonatal care following maternal SARS-CoV-2 around the time of birth infection in the UK by maternal intensive care status.

	Neonatal care following maternal SARS-CoV-2 around the time of birth	Neonatal care following maternal critical care	Neonatal care following maternal non-critical
Number	111	28^a^	76^a^
Condition at birth			
5 min Apgar <7	14 (13%)	9 (32%)	5 (7%)
10 min Apgar <7	4 (4%)	3 (11%)	1 (1%)
Intubated	14 (13%)	9 (32%)	5 (7%)
Chest compressions	2 (2%)	1 (4%)	1 (1%)
Resuscitation drugs	2 (2%)	2 (7%)	0 (0%)
Highest level of care			
Special care	18 (16%)	3 (11%)	14 (18%)
High dependency care	53 (60%)	8 (29%)	44 (58%)
Intensive care	40 (36%)	17 (61%)	18 (24%)
Highest respiratory support			
Mechanical ventilation	30 (27%)	15 (54%)	12 (16%)
Non-invasive ventilation	39 (35%)	7 (25%)	29 (38%)
Supplemental oxygen	7 (6%)	1 (4%)	6 (8%)
Neurological condition			
Seizures	3 (3%)	2 (7%)	1 (1%)
Therapeutic hypothermia	4 (4%)	1 (4%)	3 (4%)
Neonatal outcome			
Discharged home	105 (95%)	26 (93%)	73 (96%)
Still admitted	2 (2%)	1 (4%)	1 (1%)
Died	3 (3%)	1 (4%)	2 (3%)
Missing	1 (1%)	0 (0%)	0 (0%)
Breastfeeding at discharge			
Any	37 (33%)	7 (25%)	30 (39%)
Exclusive	18 (16%)	3 (11%)	15 (20%)

^a^Maternal intensive care status was missing for 7 babies.

The large majority (25/28, 89%) of mothers requiring intensive care had a SARS-CoV-2-indicated caesarean birth, compared to 11% (8/76) for mothers not receiving intensive care. Twenty-six (93%) babies born to mothers requiring intensive care were preterm compared to 43% (33/76) of babies born to mothers who were less unwell or asymptomatic (Table 1). Seven (88%) of eligible mothers requiring intensive care were given magnesium sulfate but only 8/14 (44%) were recorded as having received a full course of antenatal steroids. Only three babies born to mothers who received intensive care were exclusively breastfed at discharge from the NNU. Apgar score was lower in babies born to mothers receiving intensive care (Table 2). A total of 61% (17/28) of babies born to mothers who received intensive care required neonatal intensive care and 54% (15/28) of these babies received mechanical ventilation. Short-term outcomes at neonatal discharge were similar between babies born to mothers who received intensive care and those who did not (Table 2).

## Discussion

Using national population-level data collected through active surveillance, we found that babies born to a mother with confirmed SARS-CoV-2 infection around birth, made up a low proportion of total neonatal admissions during the first ‘wildtype’ wave of the pandemic. On average 2 per 1000 neonatal admissions in this wave of the pandemic in the UK were babies of mothers infected with perinatal SARS-CoV-2 infection;^13^ but there was variation throughout the year in the number of babies affected by maternal SARS-CoV-2 with a considerably higher proportion of neonatal admissions related to maternal SARS-CoV-2 in March and April 2020. This trend follows the epidemiology of community cases of SARS-CoV-2 in the UK, with cases rising in March and reaching a peak in April.^14^ it is not possible to determine from available data the degree to which this early increase in neonatal admissions additional may be related to additional precautions rather; however, this would be unlikely as national United Kingdom guidance from the start of the pandemic was to keep babies born to SARS-CoV-2-infected mothers with their mother when clinically possible. A total of 640 women with symptomatic SARS-CoV-2 infection gave birth during the study period; further details on maternal and perinatal care and outcomes have previously been described:^11^ hospitalised women with symptomatic were more likely to be admitted to intensive care but the absolute risk of poor outcomes was low. Neonatal data presented here support this that mortality in babies born to mothers with SARS-CoV-2 infection around birth was low with none of the three neonatal deaths directly attributed to SARS-CoV-2 infection in mother or baby.

Despite these apparently reassuring short-term outcomes, the longer-term implications of maternal SARS-CoV-2 around the time of birth may be more concerning. The rate of very preterm birth, which is strongly associated with long-term morbidity and later mortality, was 2.6% among all mothers with known perinatal SARS-CoV-2 infection who were in hospital, which is approximately double the UK national rate.^9,15^ Four term babies born to mothers with perinatal SARS-CoV-2 infection received therapeutic hypothermia for hypoxic ischaemic encephalopathy; numbers were low and preclude further formal analysis. Hypoxic ischaemic encephalopathy was not significantly more common in babies born to mothers with SARS-CoV-2 infection in pregnancy in Sweden, with only 3 cases seen among 2323 babies.^6^

This work highlights the importance of collaborative working between teams caring for pregnant women in intensive care and the maternity team.^16^ The low rate of administration of a full course of antenatal steroids to mothers receiving intensive care may be explained in part by the urgency of delivery, but is nevertheless concerning.

It is important that SARS-CoV-2 infection is recognised as a significant cause of maternal intensive care admission, particularly in unvaccinated mothers, and appropriate measures are put in place to optimise maternal and neonatal outcomes.^1^ The low rates of exclusive breastfeeding at neonatal discharge in mothers receiving intensive care are likely related to multiple factors including maternal ill-health and underline the need to follow national guidance to support breastfeeding and expression of breastmilk among mothers who are receiving intensive care.^17^ The well-established benefits of breastfeeding, including potential transfer of passive immunity against SARS-CoV-2 to the neonate, comprehensively outweigh any unproven risk of viral transmission.^18^

In keeping with studies in other age groups and populations in the first pandemic wave,^3^ babies born to mothers from black, Asian, and mixed or other ethnic groups were over-represented in this cohort.^19^ This has been attributed to socioeconomic differences and compounded by the impact of systemic structural and social inequity.^20,21^

The major strength of this study is that it reports UK-wide, population-level data from a national health service collected through an established active surveillance system with high reporting levels, linkage with national obstetric active surveillance to ensure high case ascertainment and the addition of routinely recorded neonatal data held in the NNRD. A weakness of the study is that the true incidence of SARS-CoV-2 infection within 7 days of birth is not known because national testing of all obstetric admissions was only introduced part way through this study in May 2020.

Other limitations include the absence of both longitudinal data on laboratory test results and results of neonatal imaging which may inform prognosis. Neither do we have outcomes for babies not admitted to an NNU. The long-term effects of early life exposure to SARS-CoV-2 are unknown, and ongoing data collection, linkage and follow-up are crucial.

## Conclusions

Extremely preterm birth was more common among mothers with SARS-CoV-2 infection around the time of birth, but babies born to mothers with perinatal SARS-CoV-2 infection made up a low proportion of UK NNU admissions over the first 6 months of the UK pandemic and neonatal SARS-CoV-2 infection was uncommon in these babies.

## Supplementary information

Supplemental material

## Data Availability

The datasets generated during and analysed during the current study are available from the corresponding author on reasonable request.
