# Supplementary material for: Neonatal outcomes of maternal SARS-CoV-2 infection in the UK: a prospective cohort study using active surveillance
Source: Pediatr Res. 2023 Mar 10;94(3):1203–8. doi: 10.1038/s41390-023-02527-z (PMC10000338; doi:10.1038/s41390-023-02527-z)
Supplement: Supplementary file 1 — Supplemental material [file 41390_2023_2527_MOESM1_ESM.pdf]

Supplemental data: UK neonatal outcomes of maternal SARS-CoV-2 infection: a prospective cohort study using active surveillance

Contents

	page
Details of the Neonatal complications of COVID-19 Collaborative Group	2
Acknowledgements	7

Details of the Neonatal complications of COVID-19 Collaborative Group

Writing committee

Shohaib Ali MBBS[a], Helen Mactier MD[b], Alessandra Morelli MSc[c], , Madeleine Hurd BSc[b], Anna Placzek MA[b], Marian Knight DPhil[b], Shamez Ladhani PhD[d], Elizabeth S Draper PhD[e], Don Sharkey PhD[f], Cora Doherty MD[g], Jennifer J Kurinczuk MD[c], Maria A Quigley MSc[c], Chris Gale PhD[h]

[a] Academic Foundation Year Trainee, Imperial College London, School of Public Health, Faculty of Medicine, Chelsea and Westminster campus, 4th Floor, Lift Bank D, 369 Fulham Road, SW10 9NH, UK

[b] Princess Royal Maternity and the University of Glasgow, Glasgow, UK

[c] NIHR Policy Research Unit in Maternal and Neonatal Health and Care, National Perinatal Epidemiology Unit, Nuffield Department of Population Health, University of Oxford, UK

[d] Public Health England, Colindale, UK; Reader, St. George's University of London, UK

[e] Department of Health Sciences, University of Leicester, Centre for Medicine, University Road, Leicester, UK

[f] Academic Child Health, School of Medicine, University of Nottingham, UK

[g] University Hospital of Wales, Cardiff, UK

[h] Professor of Neonatal Medicine, Imperial College London, School of Public Health, Faculty of Medicine, Chelsea and Westminster campus, 4th Floor, Lift Bank D, 369 Fulham Road, SW10 9NH, UK

Reporting clinicians

Good Hope Hospital: Dr Babi Rani Pal; **Aberdeen Maternity Hospital**: Dr Lambrini Psiouri; **Aberdeen Royal Infirmary**: Dr Saulius Satas, Dr Catriona Middleton; **Addenbrooke's Hospital**: Dr Sajeev Job; Adelaide House: Dr Melanie Douglas; **Airedale General Hospital**: Emma Dooks, Dr Philippa Rawling ; **Alder Hey Children's Hospital**: Dr Andrew Riordan, Dr Narayani Vayyeti, Dr Clare Pain, Dr David Porter, Dr Stephen McWilliam, Dr Charlotte Durand; **Alexandra Hospital**: Dr Tom Charles Dawson; **Altnagelvin Area Hospital**: Dr Damian Armstrong, Dr Mary Ledwidge; **Antrim Area Hospital**: Dr Lynne McFetridge; **Arrowe Park Hospital**: Dr Anand Kamlanathan, Dr Sarah Thompson, Dr David Lacy; **Barking Hospital**: Ms Helen Smith; **Barnet Hospital:** Dr Shanthi Shanmugalingam, Dr Esther Freeman; **Basildon University Hospital:** Dr Donna Southam, Dr Sanjay Rawal; **Bedford Hospital**: Dr Jennifer Valentine; **Birmingham Children's Hospital:** Dr Divya Gurudutt, Dr Harsha Gowda, Dr Sarah Denniston, Dr Victoria Fradd, Dr Vidya Garikapati, Dr Amy Walker, Dr Pinki Surana; **Birmingham Women's Hospital:** Dr Manobi Borooah, Dr Gergely Toldi, Dr Matthew Nash; **Bradford Royal Infirmary:** Dr Liz Ingram, Dr Sam Wallis, Dr Sam Oddie, Dr Chris Day, Dr Rebecca Newbegin, Dr Firth; **Bradford Teaching Hospitals:** Dr Ellen Mosley, Dr Chakrapani Vasudevan; **Breightmet Health Centre:** Dr Gabrielle Lipshen; **Bristol Royal Hospital for Children:** Dr Stefania Vergnano, Dr Jeyesh Patel, Dr Marion Ruth Roderick, Dr Frances Hutchings, Dr Hannah Langford-Wood, Dr Malini Ketty, Dr Hester Taekema; **Bronglais General Hospital:** Dr Alzbeta Kolenova; **Broomfield Hospital:** Dr Dean Richard Lethaby, Dr Rachel Thomas; **Burnley General Hospital:** Dr Amitava Sur; **Calderdale Royal Hospital:** Dr Matthew Taylor, Dr David Bromley, David Bromley; **Cavan General Hospital:** Dr Alan Finan; **Central Middlesex Hospital:** Dr Ashiya Ali; **Chelsea & Westminster Hospital:** Dr Hester Yorke, Dr Catherine O'Sullivan, Dr Deena-Shefali Patel, Dr Nora Tusor, Dr Walton D'Costa, Dr Sabita Uthaya,

Dr Cheryl Battersby, Dr Mark Thomas; **Chesterfield Royal Hospital:** Dr Penelope Young; **Children's University Hospital - Dublin:** Dr Michael Riordan; **City Hospital & Birmingham Treatment Centre:** Dr Penelope Broggio, Dr Lindsay Halpern, Dr Sheilah Kamupira; **Colchester General Hospital:** Dr Joakim Anderson; **Conquest Hospital:** Dr Manivannan Kandasamy; **Countess of Chester Hospital:** Dr Ravi Jayaram, Dr Stephen Paul Brearey, Dr Helen Dallow, Dr Joanne Marie Dangerfield, Dr Alison Timmis, Dr Victoria Guratsky, Dr S Murthy Saladi; **Craigavon Area Hospital:** Dr Veena Vasi, Dr Lesley-Ann Funston, Dr David George Grier, Dr Philip Quinn, Dr David Graham; **Croydon University Hospital:** Dr Grant Marais, Dr John Chang, Dr Arun Kumar; **Darent Valley Hospital:** Dr Abdul Hasib; Darlington Memorial Hospital: Dr John Furness; **Derbyshire Children's Hospital:** Dr Jennifer Evennett, Dr Richard Bowker, Dr Velur Palaniswamy Balasubramaniam, Dr Claire Weights; Dr Gisela Robinson, Dr Anneli Wyn-Davies; **Derriford Hospital:** Dr Oladipo Aworinde, Dr Georgina Selby; **Diana Princess of Wales Hospital:** Dr Bemigho Etuwewe; **Dorset County Hospital:** Dr Dominic Sheehy; **Dumfries & Galloway Royal Infirmary:** Dr Andrew Eccleston; **Ealing Hospital:** Dr Ewa Lichtarowicz-Krynska; **East Surrey Hospital:** Dr Bindu Nair Radha, Dr Lola Adenuga; Epsom General Hospital: Dr Saifa Rashid; **Evelina Children's Hospital:** Dr Emma Parish, Dr Claire Lemer, Dr Ella Aidoo, Dr Chloe Macaulay; **Forth Valley Royal Hospital:** Dr Kristyna Bohmova, Dr Dominic O'Reilly; Dr Sabine Grosser; **Frimley Park Hospital**: Dr Sanjay Jaiswal; **Furness General Hospital:** Dr Ashutosh Kale; **Glan Clwyd Hospital:** Dr Sandra Bakker, Dr Oliver Rackham, Dr Amanda McKenna, Dr Lee Wisby; **Gloucester Royal Hospital:** Dr Miles Wagstaff, Dr Miles Wagstaff; **Good Hope Hospital:** Daniel Dogar; Grange University Hospital: Dr Gillian Smith; **Great North Children's Hospital:** Dr Andrew Ian Villis, Dr Jason Gane; **Great Ormond Street Hospital:** Dr Alasdair Bamford, Dr Mark Peters, Dr Doris Abomeli, Dr Wesley Hayes, Dr Cho Ng; **Great Western Hospital:** Dr Sarah Bates, Dr Claire Broomfield; Hereford County Hospital: Dr Simon Meyrick, Dr Cathryn Seagrave; **Hillingdon Hospital:** Dr Tristan Bate, Dr Elizabeth Lek; Dr Alex CHAN, Dr Jide Menakaya, Dr Devangi Thakkar, Dr Jaikumar Ganapathi; **Hinchingbrooke Hospital:** Dr Hilary Dixon, Dr Philip Gauci; Homerton Hospital: Dr Marianna Varsami, Dr Julia Thomson, Dr Ravi Prakash, Dr Claire Howarth, Dr Sujith Pereira;

Huddersfield Royal Infirmary: Dr Karin Schwarz, Dr Salamiah Burgess; **Hull Royal Infirmary:** Dr Hilary Klonin, Dr Hani Khdir, Dr Aparna Manou, Dr Verghese Mathew; **Imperial College School of Medicine:** Dr Simon Nadel, Dr Aubrey Cunnington; **Ipswich Hospital:** Dr Matthew James; **James Cook University Hospital:** Dr Shalabh Garg; **James Paget University Hospital:** Dr Priyadarshan Ambadkar, Dr John Chapman; **Jersey General Hospital:** Dr David Lawrenson; **Jessop Wing Hospital:** Dr Elizabeth Pilling, Dr Porus Bustani; **John Radcliffe Hospital:** Dr Eleri Adams, Dr Dominic Kelly, Dr Charles Roehr; **Joyce Green Hospital:** Dr Selwyn D'Costa; **Kettering Children's Hospital:** Dr Pratibha Rao, Dr Keshavamurthy Kallambella Sushilendra; **King George Hospital:** Dr Morgan Keane; **Kings College Hospital:** Dr Theodoros Dassios, Dr Sreena Das, Dr Lucy Pickard, Dr Zainab Kassim; **King's Mill Hospital:** Dr Rebecca Sands, Dr Simon Rhodes; **Kingston Hospital:** Dr Matthew Lee, Dr Edit Molnar, Dr Unice Tawiah Naakai Nartey, Dr Jon Filkin, Dr Nader Abd El Twab Elgharably; **Leeds General Infirmary:** Dr Sian Cooper, Dr Ramesh Kumar, Dr Kerry Jeavons, Dr Elizabeth Evans, Dr Christopher Forster, Dr Amelia Shaw, Dr Elizabeth McKechnie, Dr Anne-Marie Childs, Dr Elizabeth Day, Dr Rachel Toone, Dr Joanna Wright, Dr Sharon English, Dr Nicola Mullins; **Leicester General Hospital:** Dr Gareth Lewis; Leicester Royal Infirmary: Dr Premkumar Sundaram; Dr Habab Mekki, Dr Andrew Currie, Dr Jonathan Cusack, Dr Vikas Saxena, Dr Joe Fawke, Dr Jane Gill, Dr Kamini Yadav, Dr Mohammad Zoha, Dr Joanna Behrsin, Dr Vinayak Rai, Dr Robin Miralles, Marie Hubbard, Dr Nicola Owen, Dr Usha Niranjan; **Liverpool Women's Hospital:** Dr Richard Hutchinson; **Luton & Dunstable Hospital:** Dr Amy Carmichael, Dr Doris Iyamabo, Dr Jennifer Birch; **Maidstone Hospital:** Dr Siaw Chieng, Dr Laura J Louise Halpin; Manor Hospital: Dr Rayasandra Gireesh, Dr Raghu Krishnamurthy, Dr Ashok Karupaiah, Dr Pooja Shivananda Siddhi; **Medway Maritime Hospital:** Dr Ghada Ramadan, Dr Santosh Pattnayak ;

Milton Keynes General Hospital: Dr Zuzanna Gawlowski, Dr D Gonapoladeniya, Dr Indranil Misra, Dr Mya Aye; **Musgrove Park Hospital:** Dr Alexandra Powell, Dr Nicola Johnson; Nevill Hall Hospital: Dr Ravi Manikonda, Dr Yvette Cloete, Dr Nakul Gupta, Dr Marcus Pierrepoint; **New Cross Hospital:** Dr Robert Negrine, Dr Melanie Sutcliffe, Dr Buvenekaba Kumararatne, Dr Julie Brent, Dr Chrisantha Halahakoon, Dr Richard Heaver, Dr Chrisantha Halahakoo, Dr Richard Heaver, Dr Surinder Judge; **Newham Hospital:** Dr Nicolene Plaatjies, Dr Susan Liebeschuetz, Dr Esmira Jafarova, Dr Nicolene Plaatjies, Dr Imdad Ali, Dr Ivone Lancoma-Malcom, Dr Rakesh Ravi; **Ninewells Hospital and Medical School:** Dr Jennifer Scotland, **Noah's Ark Children's Hospital for Wales:** Dr Ruth Elizabeth Hanks; **Norfolk & Norwich Univ Hospital:** Dr Paul Clarke; Dr Catherine Thomas; Dr Priyadarsini Muthukumar; Dr Mark Dyke, Dr Florence Walston; **North Devon District Hospital:** Dr Michael Selter; **North Hampshire Hospital:** Dr Lucinda Winckworth; North Manchester General Hospital: Dr Hatem Sager; **North Middlesex Hospital:** Dr Cheentan Singh, Dr Piyusha Kapila, Dr Cassandra Gyamtso; Dr Linda Walker, Dr Fionnghuala Fuller, Dr Lesley Alsford, Dr Rosalind Mensah, Dr Janani Pallawela, Dr Olu Wilkey, Dr Bijan Shahrad, Dr Aparna Nambisan, Dr Dhruv Rastogi; **North Tyneside General Hospital:** Dr Ivonne Haar, Dr Sangeeta Tiwary; **Northampton General Hospital:** Dr Cathryn Chadwick, Dr Sathyaseelan Jayaseelan, Dr Nick Barnes, Dr Fiona Thompson, Dr Janet Collinson, Richard Breene; **Northumberland Child Health Centre:** Dr Sangeeta Tiwary; **Northwick Park Hospital:** Dr Richard Nicholl, Ms Anam Fayadh, Dr Krzysztof Zieba, Dr Edit Fukari-Irvine; **Nottingham City Hospital:** Dr Dushyant Batra, Dr Stylianie Tsilika, Dr Anushma Sharma; **Our Lady's Hospital for Sick Children:** Dr Fiona Ringholz, Dr Sinead Harty; **Peterborough City Hospital:** Dr Katharine McDevitt, Dr Mona Aslam, Dr Ramya Ramaswamy, Coralie Huson, Dr David John Hopkins, Dr Tim Jones; Dr Katharine McDevitt; **Pinderfields General Hospital:** Dr Natasha De Vere, Dr Kallinath Shyamanur, David Gibson; **Poole Hospital:** Dr Mark Tighe, Dr Peter McEwan; **Portsmouth Community:** Dr Kathy Padoa; **Princess Alexandra Hospital:** Dr Chinnappa Reddy; **Princess Anne Hospital**: Dr Victoria Puddy, Dr Rupjani Banerjee, Dr Kelly Brown, Dr Kevin Goss, Dr Helen Fielder; **Princess Elizabeth Hospital:** Dr Clare Betteridge; **Princess of Wales Hospital:** Dr Torsten Hildebrandt; **Princess Royal Maternity Hospital:** Dr Tomaz Dygas; **Princess Royal University Hospital:** Dr Stella Nzekwue; **Queen Alexandra Hospital:** Dr Huw Jones, Dr Tim Scorrer, Dr Amanda Freeman, Dr Karen Deem, Dr Borbone, Dr Roy Sievers, Dr Jennie Pridgeon; **Queen Charlotte's & Chelsea Hospital:** Dr Aniko Deierl, Dr Jayanta Banerjee; Dr Emma Porter, **Queen Elizabeth Hospital - Birmingham:** Dr Manobi Borooah; **Queen Elizabeth Hospital - East Anglia:** Dr Abigail Reeve; **Queen Elizabeth Hospital - Lewisham and Greenwich:** Dr Julie Lord, Dr Olutoyin Banjoko, Emma Gardiner; **Queen Elizabeth University Hospital, Glasgow:** Dr Ruth Bland; **Queen Mary's Hospital for Children:** Dr Daniel Langer, Dr Ralf Hartung, Dr Arunava Kundu; **Queen's Hospital - Romford:** Dr Ambalika Das, Dr Helen Smith, Dr Donna Nicholls, Dr Ranjith Joseph; **Queen's Medical Centre - Nottingham:** Dr Lleona Lee, Dr Anjum Deorukhkar, Dr Jodi Wood; **Rosie Maternity Hospital:** Dr Stergios Papakostas; **Rotheram General Hospital:** Dr Soma Sengupta; **Royal Albert Edward Infirmary:** Dr Hough; **Royal Alexandra Hospital:** Dr Hilary Conetta; **Royal Belfast Hospital:** Dr Rachel Beckett, Dr Elizabeth Dalzell; **Royal Belfast Hospital:** Dr Paul Moriarty; **Royal Berkshire & Battle Hospitals:** Dr Ahmed Aldouri, Dr Chandan Yaliwal, Dr Ravi Kumar, Dr Ann Gordon, Dr Nicola Pritchard, Dr Kementhri Naidoo; **Royal Berkshire Hospital:** Dr Syed Akmal Hussain; **Royal Blackburn Hospital:** Dr Andrew Cox; **Royal Bolton Hospital:** Dr Fiona Watson, Dr Shanmuga Sundaram, Dr Archana Mishra, Dr Jo Morgan, Dr Ian Freeman; **Royal Brompton Hospital:** Dr Piers Daubeney; **Royal Cornwall Hospital:** Dr Thomas Fontaine; **Royal Devon & Exeter Hospital:** Dr Sian Ludman, Dr Simon Parke, Dr David Mabin, Dr Nagendra Venkata, Dr Pasupulety Venkata; **Royal Free Hospital:** Dr James Rosenberg, Dr Marice Theron, Dr Eleanor M Bond; Royal Glamorgan Hospital: Dr Takin Omolokun; **Royal Gwent Hospital:** Dr Tanoj Gopalan Kollamparambil, Dr Sarmistha Maity, Dr Murali Natti, Dr Sarika Goel; **Royal Hampshire County Hospital:** Dr Lucinda Winckworth; **Royal Hospital for Children:** Dr Neil Patel, Dr Dominic Cochran, Dr Helen McDevitt, Dr Andrew Brunton, Dr Jonathan Coutts, Dr Louise Leven, Dr Jennifer

Mitchell, Dr Owen Forbes, Dr Rosie Hague, Dr Morag Nina Joyce Wilson; **Royal Hospital for Sick Children, Edinburgh:** Dr Mairi Stark; **Royal Infirmary of Edinburgh:** Dr Ewen Johnston; **Royal Jubilee Maternity Hospital:** Dr Stan Craig; **Royal Lancaster Infirmary:** Dr Clare Peckham, Dr Joanne Fedee; **Royal Oldham Hospital:** Dr Fazal Rehman, Dr Sarah McCullough, Dr Anita Vayalakkad, Zainab Sarwar, Dr Lydia Bowden; **Royal Preston Hospital:** Dr Raju Narasimhan, Dr Hyacienth Akaolisa Egbeama, Katrina Rigby, Dr Aubrey Makhalira; **Royal Stoke University Hospital:** Dr Laura Roe, Dr Olayinka Kowobari, Dr Lee Abbott, Dr Julia Uffindell; **Royal Surrey County Hospital:** Dr Ozan Hanci, Dr Diarra Greene, Dr Soad Habeeb, Dr Sameh El-Sayed Zaki Abdulsamea, Dr Catherine Garland, Dr Nikolay Drenchev, **Royal United Hospital:** Dr Tobias Hunt, Dr Steve Jones, Dr Dan Jolley, **Royal Victoria Infirmary:** Dr Robert Tinnion, Dr Julie Groombridge; Dr Stefan Zalewski, Dr Jenna Gillone, Dr R Hearn, Dr Julie Groombridge; **Russells Hall Hospital:** Dr Evans Chingwenje, Dr Samanthi Wilegoda ; **Salisbury District Hospital:** Dr Philippa Ridley; **Scunthorpe General Hospital:** Dr Rasheed Oba; **Sheffield Children's Hospital:** Dr Alison Smith, Dr Lucy Hinds, Dr Rachel Riddell, Dr Mairi Gillespie, Dr Soma Sengupta; **Singleton Hospital:** Dr Jamie Evans, Dr Geraint Morris; **South West Acute Hospital:** Dr Gerry Mackin; **Southampton General Hospital:** Dr Mark Johnson, Dr Anne-Marie Goss, Dr Helen Rutkowska, Dr Jason Michael Barling; **Southend General Hospital:** Dr Raj Gupta, Dr Jennifer Foster; **Southend University Hospital:** Jennifer Foster, Dr Vineet Gupta, Dr Ravi Chetan, Dr Veena Rao, Dr Ravi Chetan; **Southern General Hospital, Glasgow:** Dr Joyce O' Shea; **Southmead General Hospital:** Dr Claire Michelle Rose, Dr Richard Wach, Dr Faith Emery; Dr Madhavi Parvathareddy, Dr Paul Mannix; **St George's University Hospital :** Dr Sijo Francis, Dr Danielle Hake, Dr Sophie Robinson, Dr Daniel Langer; **St James University Hospital, Leeds:** Dr Kathryn Johnson, Dr Liz McKechnie; **St Mary's Hospital - London:** Dr Jayanta Banerjee, Dr Caroline Louise Scott-Lang,Dr Jenny Ziprin, Dr Geraldine Ng; **St Mary's Hospital - Manchester:** Dr Sajit Nedungadi; Dr Ruth Gottstein; Dr Kalwa Munthali; **St Peter's Hospital:** Dr Alison Groves, Dr Mayu Otsuka, Dr Vennila Ponnusamy, **St Peter's Hospital:** Dr Jennifer McGrath, Dr Maria Samantha Edwards, Dr Clare Hill, Dr Peter Martin; Dr Luciana Elisabeta Ene; **St Richard's Hospital:** Dr Ann-Marie Buckley; **St Thomas' Hospital:** Dr Timothy Watts; **Stepping Hill Hospital, Stockport:** Dr Carrie Heal; **Stoke Mandeville Hospital:** Dr Caroline Lowdon, Dr Ralph Robertson, Dr Gopa Sarkar; **Sunderland Royal Hospital:** Dr Chike Onwuneme; **Tameside General Hospital:** Dr Helen Purves, Dr David Levy, Dr Trupti Dhorajiwala, Dr Robert Block; **Tayside Children's Hospital:** Dr Birgit Wefers; **The James Cook University Hospital:** Dr Ginny Birrell, Dr Thomas Skeath, Dr Maeve O'Sullivan, Dr Helen Chitty; **The Princess Royal Hospital:** Dr Wendy Tyler; **The Princess Royal Hospital:** Dr Sanjeev Deshpande; **The Royal London Hospital:** Dr Hemmay Raychaudhuri, Dr Catherine Warrick, Dr Nicolene Plaatjies, Dr Caroline Francia, Dr Caroline May, Dr Ajay Sinha, Dr Anup Kage, Dr Anne Opute, Dr Rainer Ebel, Dr Gemma Sedgwick; **The Ulster Hospital:** Dr Julia Courtney, Dr Carl Harris, Dr Damhnait Cassidy, Dr Michael McGowan; **The York Hospital:** Dr Luke Kevin McLaughlin, Dr Rebecca Proudfoot, Dr Dominic Smith, Dr Liz Baker; **Torbay Hospital:** Dr Richard Tozer, Dr Jonathan Graham, Dr Esther J Morris, Dr Alison Janzen; **Tunbridge Wells Hospital:** Louise Swaminathan; **University College Hospital London:** Dr Sarah Eisen, Dr Christina Kortsalioudaki, Dr Andrea Leigh, Dr Leigh Dyet; **University Hospital Coventry:** Dr Karen McLachlan; **University Hospital Crosshouse:** Dr Nuno Cordeiro, Dr Althaf Ansary; **University Hospital Lewisham:** Dr Ozioma Obi, Dr Neha Sharma, Dr Kumudini Gomez, Dr Emma Gardiner: **University Hospital of North Tees:** Alex Ramshaw; **University Hospital of Wales:** Dr Nitin Goel, Dr Amarkumar Asokkumar, Dr Marcia Scheller, Dr Elisa Smit, Dr Cora Doherty; **University Hospital Wishaw:** Dr Augusta Anenih, Dr Hatice Isikli, Dr Padma Rajagopal, Dr Caroline Delahunty, Dr Adrienne Sullivan; **University Hospitals Dorset NHS Foundation Trust:** Dr Amy Roff; **University Hospitals of Leicester NHS Trust:** Dr Deepa Panjwani; **Victoria Hospital - Blackpool:** Dr Mohammed Idris Ahmed, Dr Christopher John Rawlingson, Prof Morris Gordon; **Warrington Hospital:** Dr Delyth Webb, Dr Colin Wong, Dr Rachael Sutton, Dr Elinor Thomason, Dr Delyth Webb; **Warwick Hospital:** Dr Sumedha Chamalie Bird, Dr Kate Blake; **Watford General Hospital:** Dr CS Narayanan, Dr Nirmala Costa-Fernandes,

Dr Nazakat Merchant, Dr Renton L'Heureux, Dr Avinash Jinadatha, Dr Meera Mallya; **West Cumberland Hospital:** Dr Clive Graham, Dr Hannah Holt-Davis; **West Middlesex University Hospital:** Dr Tsitsi Dadirai Chawatama, Dr Eleanor Hulse; **West Suffolk Hospital:** Dr Ian Evans; **Wexham Park Hospital:** Dr Kanaga Raj Sinnathuray, Dr Sujata Narayan Edate; **Whipps Cross Hospital:** Dr Nicolene Plaatjies, Dr John Ho; **Whittington Hospital:** Dr Juliet Penrice, Dr Andrew Robins, Dr Alka Desai, Dr Gopa Sen, Dr Caroline Fertleman, Dr Nischal Rao; William Harvey Hospital: Dr Amit Gupta, Dr Vimal Vasu; **Worcestershire Royal Hospital**, Dr Subramania Kalambettu, Dr Jessie Brain, Dr Viviana Anne Sophie Weckemann; **Worthing Hospital:** Dr Gillian Hobden, Dr Stuart Nicholls, Dr Jonathan Rabbs; **Wycombe General Hospital**: Dr Boon Tang; **Wythenshawe Hospital**: Dr Asim Ahmed, Dr Ahmed Elazabi, Dr Abhijeet Godhamgaonkar; **Ysbyty Gwynedd District General Hospital, Bangor**: Dr Shakir Saeed

British Paediatric Surveillance Unit, Royal College of Paediatrics and Child Health: Richard Lynn, Jacob Avis, Farhana Ahmed

Public, parent and patient involvement: Charlotte Bevan and Rachel Plachcinski, **PPPI Leads Policy Research Unit in Maternal and Neonatal Health and Care**, who commented on the design, protocol and the public facing materials.

Acknowledgements

We would also like to acknowledge the following groups who worked in extraordinary circumstances to expedite the process of getting this study set up within three weeks. Without their support and dedication, often working out of hour generally working from home, this would not have been possible to achieve.

British Paediatric Surveillance Unit Scientific Committee

Confidentiality Advisory Group, Health Research Authority

Health Protection Scotland

Health Research Authority

Information Governance team, Nuffield Department of Population Health, University of Oxford

Information Services Division, Scotland

Members of the MBRRACE-UK third sector stakeholder group

Multicentre Research Ethics Committee

Northern Ireland Maternal and Child Health, Public Health Agency

Policy Research Programme, Department of Health and Social Care, England

Public Benefit and Privacy Panel for Health and Social Care, Scotland

Public Health England

Sponsors, Clinical Trials and Research Governance, Research Support, University of Oxford
